# Dietary supplementation with 15% tomato pomace (*Solanum lycopersicum* L.) improves sperm production and antioxidant status in aged male broiler breeders

**DOI:** 10.1016/j.psj.2024.104553

**Published:** 2024-11-22

**Authors:** Amir Mosayyeb Zadeh, Seyyed Ali Mirghelenj, Mohsen Daneshyar, Mohsen Eslami, Mohammad Amir Karimi Torshizi, Mahdi Zhandi, Touba Nadri, John Patrick Kastelic, Peyman Hasanloo, Mehdi Nabiloo

**Affiliations:** a Department of Animal Science, College of Agriculture, Urmia University, Urmia, Iran; b Department of Theriogenology, Faculty of Veterinary Medicine, Urmia University, Urmia, Iran; c Department of Poultry Science, Faculty of Agriculture, Tarbiat Modarres University, Tehran, Iran; d Department of Animal Science, Faculty of Agriculture, University College of Agriculture and Natural Resources, University of Tehran, Iran; e Faculty of Veterinary Medicine, University of Calgary, Calgary, Alberta, Canada

**Keywords:** Arginine, Gene expression, Male broiler breeder, Testosterone, Tomato pomace

## Abstract

The objective was to investigate effects of dietary tomato pomace (**TP**) and L-Arginine (L-Arg; 10% above nutritional recommendation) in aged male broiler breeders. Thirty male broiler breeders (Ross 308), 58 wk old, were randomly and equally allocated into five experimental groups: basal diet (**C**); 5% TP-supplemented (**T-5**); 10% TP-supplemented (**T-10**); 15% TP-supplemented (**T-15**); and L-Arg supplemented (**L-10**) groups. In contrast to the Control and TP-supplemented groups, sperm of L-10 roosters had higher C14:1, C16:0 and C18:1 n-9c proportions, total saturated fatty acid (**FA**) contents, and n-6:n-3 ratios, but a lower C22:4 n-6 content (*P*<0.05). However, sperm of T-15 roosters had increased C18:2 n-6, C22:6 n-3 and total unsaturated FA contents but reduced n-6:n-3 ratios compared to Control birds (*P*<0.05). Additionally, serum of T-15 roosters had lower cholesterol and triglyceride (**TG**) concentrations, along with decreased aspartate aminotransferase (**AST**) activity (*P*<0.05). In serum of T-15 and L-10 roosters, there were increases (*P*<0.05) in total antioxidant capacity (**TAC**) and uric acid (**UA**) and a reduction (*P*<0.05) in malondialdehyde (**MDA**) concentration. The T-15 roosters had higher (*P*<0.05) serum testosterone concentration and testes glutathione peroxidase (**GPx**) activity. Superoxide dismutase (**SOD**) content was reduced (*P*<0.05) in L-10 roosters. Seminiferous tubule diameter (**STD**) and seminiferous epithelium thickness (**SET**) were increased in all groups fed TP (*P*<0.05) and T-10 and T-15 roosters had increases (*P*<0.05) in tubular differentiation indices (**TDI**). The T-15 and L-10 groups had higher mRNA expressions of *AKT1* and *Nrf2* (*P*<0.05). In conclusion, in aged male broiler breeders, up to 15% TP supplementation is recommended to improve reproductive performance by enhancing testes histology and antioxidant status.

## Highlights

• T-15 increased total PUFAs and reduced n-6/n-3 content in sperm FA profile.
• T-15 improved testes' antioxidant status by increasing expression of *Nrf2*.
• T-15 increased testes’ histological indices by increasing expression of *AKT1*.
• L-10 treatment reduced histological indices and antioxidant status of testes.

## Introduction

Supplementing diets of broiler breeder roosters with tomato pomace (TP) or lycopene (dominant carotenoid in red fruits) enhanced reproductive performance by improving semen characteristics, including greater sperm concentration and more live and fewer abnormal sperm, attributed to the unique antioxidant property of lycopene (Mangiagalli et al., 2010; Rosato et al., 2012; Saemi et al., 2012). Those findings were verified in our recent work with aged commercial male broiler breeders fed TP-supplemented diets (Mosayyeb Zadeh et al., 2023); however, specific mechanisms remain unknown. In addition to confirming the findings of Saemi et al. (2012), our recent experiment (Mosayyeb Zadeh et al., 2023) indicated that 15% dietary TP supplementation may improve fertility of old roosters experiencing reproductive system deterioration due to oxidative stress, e.g., due to aging (Beckman and Ames, 1998).

Tomato pomace (*Solanum lycopersicum* L.) contains a vast range of biochemical compounds (including phytochemicals, flavonoids, and polyphenols) reported to positively influence reproductive performance of male broiler breeders by altering sperm FA profile and blood biochemistry, especially testosterone concentrations, through their antioxidant property. Furthermore, L-Arg supplementation increased blood testosterone in roosters and may affect testes weight and histology (Abbaspour et al., 2019; Ahangar et al., 2017). Perhaps selenium and vitamin E (alone or in combination) improve testes histology through their antioxidant property (Khalid et al., 2016; Sabzian-Melei et al. 2022). Furthermore, lycopene may up-regulate antioxidant genes, e.g., nuclear factor-erythroid 2-related factor 2 (**Nrf2**) (Gum and Cho, 2013; Taguchi et al., 2011). Therefore, like α-linoleic acid (Ye et al., 2021), it is assumed that stimulatory effects of lycopene on expression of the AKT serine or threonine kinase one gene (***AKT1***; has a role in spermatogenesis), may enhance roosters’ reproductive performance by improving semen characteristic traits (especially sperm concentration).

As an essential amino acid (**AA**) (Tamir and Ratner, 1963), L-Arg has important effects on reproductive performance of poultry, particularly roosters. According to various studies, L-Arg and its derivatives enhance rooster fertility and egg hatchability (Tapeh et al., 2017), improve sperm penetration to the blastoderm layer (Sharideh et al., 2016), improve sperm forward motility (Lefièvre et al., 2007; Tapeh et al., 2017), increase blood testosterone concentration (Alhaj et al., 2018; Altawash et al., 2017), semen volume (Ahangar et al., 2017), and TDI, sperm repopulation index, and spermatogenesis in testicular histology (Abbaspour et al., 2019; Ahangar et al., 2017) primarily due to its antioxidant properties, which are linked to nitric oxide production, and its energy-boosting effects, attributed to its contributions to guanidinoacetic acid formation.

Exact mechanisms of improved fertility rate and semen characteristics in male broiler breeders fed TP or lycopene are not clear. Therefore, the objective was to investigate effects of dietary TP (5 to 15%) and L-Arg (10% above nutritional recommendation) in aged male broiler breeders. Sperm FA profile, blood and testicular biochemistry, and testicular histology and gene expression of roosters from our previous study (Mosayyeb Zadeh et al., 2023) were examined.

## Materials and methods

This study was reviewed and approved by the Animal Care Committee and Animal Research Ethics Board, Department of Animal Science, Urmia University, Iran (Protocol IR-UU-AEC 1344/PD/3).

### Chemicals

All chemicals used were from either Merck Co. (Darmstadt, Germany) or Sigma Aldrich Co. (St. Louis, MO, USA).

### Birds and management

Thirty male broiler breeders, 58 wk old (Ross 308) were procured from a nearby farm and maintained under controlled environmental conditions (20 ± 1.5 °C and 40 to 50% relative humidity) in individual floor pens (120 × 120 cm) for 12 consecutive weeks, with the first 2 weeks considered an adaptation period. Thereafter, birds were randomly and equally assigned to five treatments: a) C (Basal diet), b) T-5, c) T-10, d) T-15, and e) L-10 (Table 1). Experimental diets were based on corn and soybean meal, formulated according to strain-specific nutritional recommendations, and fed in mash form, using the once-a-day feeding system (150 g/bird/day) with *ad libitum* access to fresh water.

**Table 1:** Ingredients and chemical composition of diets fed to aged broiler breeder roosters.

Ingredient (%)	C	L-10	T-5	T-10	T-15
Corn	67.51	67.52	65.90	64.32	62.74
Wheat bran	22.88	22.76	20.49	17.86	15.22
Soybean meal (39%)	6.32	6.37	5.49	4.75	4.01
Tomato pomace	–	–	5.00	10.00	15.00
DCP	1.25	1.25	1.22	1.19	1.17
CaCO_3_	0.95	0.95	0.98	1.01	1.04
Common salt	0.37	0.37	0.32	0.27	0.22
Vitamin premix^a^	0.3	0.3	0.3	0.3	0.3
Mineral premix^b^	0.3	0.3	0.3	0.3	0.3
DL-methionine (99%)	0.09	0.09	–	–	–
L-lysine (74.42%)	0.03	0.03	–	–	–
L-arginine (99 %)	–	0.06	–	–	–
Calculated nutrient content					
AMEn (kcal/kg)	2700	2700	2700	2700	2700
CP (%)	12.00	12.00	12.00	12.00	12.00
CF (%)	4.46	4.45	5.80	7.12	8.44
Calcium (%)	0.70	0.70	0.70	0.70	0.70
Available phosphorus (%)	0.35	0.35	0.35	0.35	0.35
Sodium (%)	0.17	0.17	0.17	0.17	0.17
Chloride (%)	0.26	0.26	0.33	0.41	0.48
Methionine^c^ (%)	0.30	0.30	0.30	0.32	0.34
Methionine + cystine^c^ (%)	0.53	0.53	0.59	0.60	0.61
Lysine^c^ (%)	0.50	0.50	0.57	0.66	0.76
Threonine^c^ (%)	0.41	0.41	0.44	0.47	0.49
Arginine^c^ (%)	0.68	0.74	0.83	0.97	1.11
DCAB	153.30	153.16	143.44	133.31	123.17
Lycopene^d^ (μg/g)	ND	ND	0.68	1.02	1.53

Diets (fed for 10 wk) were as follows: Basal diet (C), 5% tomato pomace supplemented (T-5), 10% tomato pomace supplemented (T-10); 15% tomato pomace supplemented (T-15), diet supplemented with L-Arg 10% above nutritional recommendation (L-10).

*Abbreviations: DCP, dicalcium phosphate; CaCO_3_, calcium carbonate; AMEn, apparent metabolizable energy corrected for nitrogen; CP, crude protein; CF, crude fiber; DCAB, dietary cation-anion balance; ND, not detected.

a Supplied per kg of diet, 12000 IU vitamin A (retinyl acetate); 3500 IU vitamin D3 (cholecalciferol); 100 IU vitamin E (DL-α-tocopheryl acetate); 5 mg vitamin K3 (menadione); 12 mg vitamin B2 (riboflavin); 3 mg vitamin B1 (thiamin); 13 mg vitamin B5 (D-pantothenic acid); 2 mg vitamin B9 (folic acid); 6 mg vitamin B6 (pyridoxine); 0.03 mg vitamin B12 (cobalamin); 0.55 mg vitamin B7 (biotin).

b Supplied per kg of diet, 120 mg Mn (MnSO_4_.H_2_O); 110 mg Zn (ZnO); 50 mg Fe (FeSO_4_.7H_2_O); 10 mg Cu (CuSO_4_.5H_2_O); 2 mg iodine (KI), 0.3 mg Se.

c Values are standardized ileal digestibility [AMINODAT 4.0 (Evonik Industries, 2010)].

d Lycopene values represent the mean lycopene contents of three samples per experimental diet.

Nutrient composition of the TP used was considered as follows: AMEn, 2,072 kcal/kg (Mhbaghy et al., 2018); CP, 16.83% (%DM); CF, 34.00% (%DM); ether extract, 8.80% (%DM); methionine + cysteine, 0.90%; methionine, 0.70%; lysine, 2.72%; arginine, 4.00%; threonine, 1.13%; tryptophan, 1.40%; asparagine, 6.84%; calcium, 0.01%; phosphorus, 0.27%; sodium, 0.42%; chloride, 2.12%; potassium 0.74%; etc. (Mosayyeb Zadeh et al., 2023). More details regarding the nutrient composition of TP are reported in Mosayyeb Zadeh et al. (2023). The lycopene content of each experimental treatment was determined using a UV-visible double beam spectrophotometer, as described in our previous study. Based on the AA content of the TP, no further AA supplementation was done.

### Feed and sperm FA profile determination

On Week 8, semen samples were collected by abdominal massage and placed in plastic vials that were immediately immersed in liquid nitrogen. Feed and semen samples were transferred to the University of Applied Science and Technology (Urmia, Iran) for FA determination, as described (AOAC, 2000a,b; 2003; Barthet et al., 2002). For feed samples, total lipids were extracted using a 2:1 (v:v) chloroform:methanol mixture, as recommended (Floch, 1957). Semen samples were thawed at 18 °C and centrifuged at 2700 g for 10 min to isolate sperm. Samples were saponified by adding 3 ml of 2M methanolic potassium hydroxide (Merk Co., Germany) and converted to methyl ester by adding 5 ml of 12% v/v methanolic sulfuric acid (Merk Co., Germany). The obtained methyl ester of FAs was extracted using 0.1 ml of heptane normal and 1 μl of the normal heptane phase injected into a Gas Chromatography (GC) machine (Agilent 6890, Agilent CO, USA), well equipped with a capillary injection valve, FA specified capillary column (Stabil-wax; 30 m long, 0.32 mm internal diameter, 0.25 μm stationary phase width), and flame ionized detector (FID). The initial temperature of the oven was 75 °C for 1 min and then increased (25 °C/min) to 240 °C (and maintained for 8 min). During the process, a flow rate of 1 and 45 ml/min was used for Nitrogen gas (as a carrier with a purity of 99.999%, from Oxygen Sabalan Co. [the UK Air Product Company's agent]) and the assembler, respectively. Injector and detectors’ temperatures were set at 250 and 280 °C, respectively. Finally, the FA profile was determined by comparing retention times belonging to the sample with that of the standard, obtained by injecting a mixture of the FA standard of all desired FAs (Sigma Aldrich). Data were analyzed using Chemstation software in the Windows environment and results reported in Table 2 (feed FA profile) and Table 3 (semen FA profile). Chloroform, normal heptane, and methanol solvents were from Caledon Co. (Canada) and used without repurification.

**Table 2:** Fatty acid profile (% of total FAs) of feed samples (*n* = 3).

Fatty acid (%)	C*	T-5	T-10	T-15	L-10
Myristic acid (C14:0)	2	2	3	3.2	5
Palmitic acid (C16:0)	15.68	15.13	16.88	15.9	16.83
Stearic acid (C18:0)	1.42	1.46	1.11	1.44	1.46
Oleic acid (C18:1 n-9)	25.47	25.76	26.05	25.84	23.37
Linolelaidic acid (C18:2 n-6t)	1.02	0.77	0.75	1.04	1.30
Linoleic acid (C18:2 n-6)	51.97	52.82	51.49	51.24	52.04
Linolenic acid (C18:3 n-3)	1.65	1.72	1.84	1.67	2.10

⁎ Diets (fed for 10 wk) were as follows: Basal diet (C), 5% tomato pomace supplemented (T-5), 10% tomato pomace supplemented (T-10); 15% tomato pomace supplemented (T-15), diet supplemented with L-Arg 10% above the nutritional recommendation (L-10). ^†^Only FAs in this table were successfully detected in test diets; remaining FAs were either very low or not detected. ^‡^Samples were obtained at Week 8 of the experiment.

**Table 3:** Effects of dietary tomato pomace and L-Arg supplementation on sperm fatty acid profile (% of total FAs) of aged male broiler breeders.

Fatty acid (%)	C†	T-5	T-10	T-15	L-10	Pooled SEM	P-Value
Capric acid (C10:0)	2.20	1.45	2.12	1.30	1.26	0.628	0.624
Undecanoic acid (C11:0)	2.05	2.69	1.80	1.44	2.33	0.565	0.298
Lauric acid (C12:0)	2.05	2.53	2.77	1.73	2.86	0.339	0.200
Myristic acid (C14:0)	0.84	2.64	1.44	1.25	2.57	0.809	0.132
Methyl myristoleate (C14:1)	0.26^c^	0.94^b^	0.82^b^	0.32^c^	2.07^a^	0.311	0.015
Palmitic acid (C16:0)	20.44^b^	19.93^b^	20.02^b^	21.79^b^	30.85^a^	1.288	<0.01
Palmitoleic acid (C16:1)	0.72	1.05	1.54	0.82	0.88	0.692	0.573
Heptadecanoic acid (17:0)	3.18	1.29	2.08	3.43	0.80	0.995	0.319
cis-10-Heptadecenoic acid (C17:1)	ND*	1.04	1.44	ND	0.8	-	-
Stearic acid (C18:0)	ND	ND	ND	ND	7.6	-	-
Oleic acid (C18:1n-9)	19.71^b^	18.92^b^	19.98^b^	17.74^b^	25.40^a^	1.416	<0.01
Linoleic acid (C18:2 n-6)	3.59^b^	5.23^ab^	5.515^ab^	7.285^a^	3.365^b^	0.947	<0.01
y-Linolenic acid (C18:3 n-6)	2.05	1.96	3.29	2.84	1.84	0.676	0.222
alpha-Linolenic acid (C18:3 n-3)	ND	ND	ND	ND	ND	-	-
Arachidic acid (C20:4 n-6)	7.01	8.10	6.87	8.76	5.53	0.981	0.233
Docosatetraenoic acid (C22:4 n-6)	13.49^a^	13.85^a^	13.48^a^	14.77^a^	10.07^b^	0.817	0.029
Docosahexaenoic acid (C22:6 n-3)	1.83^b^	2.13^b^	3.77^a^	3.96^a^	1.59^b^	0.488	<0.01
n-6:n-3 ratio	14.20^a^	13.68^a^	8.03^b^	8.59^b^	14.45^a^	0.492	0.01
Total saturated fatty acids	42.64^b^	40.04^b^	39.88^b^	36.11^b^	50.12^a^	1.554	<0.01
Total unsaturated fatty acids	57.36^a^	59.96^a^	60.11^a^	63.88^a^	49.87^b^	1.550	<0.01

⁎ *Abbreviations:* ND, not detected; SEM, standard error of means. n-6:n-3 ratio, the ratio of omega-6 fatty acids to omega-3 fatty acids.

a ^-c^Means without a common superscript differed *P*<0.05.

† Roosters (58 wk of age; *n*=6 per treatment) were fed a basal diet (C), or a basal diet supplemented with 5, 10 or 15% tomato pomace (T-5, T-10 and T-15 respectively), or a diet supplemented with L-Arg 10% above the nutritional recommendation (L-10). ^‡^Samples were obtained at Week 8 of the experiment.

### Blood biochemical contents

During Week 10, blood samples were collected from a wing vein using 22 g x 25.4 mm needles and 5 mL plastic syringes. After blood was collected, the syringe was put at a 60° angle for 2 h at room temperature (25 °C) and then centrifuged at 5600 x g for 12 min. Serum was removed and maintained at −20 °C until transferred to the laboratory (Department of Drug Applied Research Center, Tabriz University of Medical Sciences, Tabriz, Iran) for evaluations. Serum glucose concentration was measured according to the method of glucose oxidase (GOD) catalysis reaction, using a glucose determination kit from Pars Azmoon, according to manufacturer's instructions. Briefly, following blending of serum sample or standard (10 µL) with the provided reagent (1000 µL), the mixture was incubated at 37 °C for 10 min, and then light absorbance was measured at 546 nm using an autoanalyzer (Model Technicon RA 1000, Bayer) and compared to a blank (distilled water).

Serum testosterone was quantified based on the competitive enzyme immunoassay (type seven) method using Testosterone Test System kits (Monobind Inc., AccuBind ELISA Microwells, Lake Forest, CA, USA), according to manufacturer's instructions. Briefly, the Wash Buffer content was diluted to 1000 ml using deionized water. Then, Working Substrate Solution was prepared by pouring the contents of the amber vial into the clear vial. Both prepared reagents were kept at 2 to 8 °C until the assay was started. Thereafter, 10 μL of the serum sample was pipetted into microwells, and 50 μL of the ready-to-use Testosterone Enzyme Reagent was added. Microwells were mixed by gentle swirling for 30 s and then microplates were covered and incubated at room temperature (25 °C). After 1 h, microplates were decanted and 350 μL of Wash Buffer was added. Again, the microplates were washed by decanting, and this was repeated twice more (total of three times). Thereafter, 100 μL of the Working Substrate solution was added to wells and incubated for 15 min at room temperature (25 °C), then 50 μL of the Stop solution (strong acid, 1N HCl) was added to the wells and mixed gently for 20 s. Within 30 min after adding Stop solution, light absorbance of each well at 450 nm was recorded. Absorbance values acquired for each duplicate serum reference were plotted versus the corresponding testosterone concentration using computer data reduction software for ELISA assay and results reported in Fig 3. Remaining end points (TG, cholesterol, MDA, TAC, alanine amino transferase (**ALT**), AST, total protein, albumin, and UA) were measured using kits provided by Pars Azmoon, according to manufacturers’ instructions, and described in detail in the previous study (Mosayyeb Zadeh et al., 2022).

### Testes biochemical content

After 12 wk, birds were euthanized by cervical dislocation, testes removed and samples of testicular tissue were transferred to the laboratory (Department of Drug Applied Research Center, Tabriz University of Medical Sciences, Tabriz, Iran) at −70 °C to determine TAC, GPx, protein, SOD, and MDA. Testes samples (2.5 g) were prepared by adding 1.15% KCl buffer solution (pH 7 and 1:10 ratio) and homogenizing at 5000 x g for 5 min. The resulting mixture was centrifuged at 7000 x g for 10 min (at 4 °C), and the supernatant separated and stored at −20 °C. Detailed descriptions of measuring TAC, GPx, protein, SOD, and MDA were reported in our previous study (Mosayyeb Zadeh et al., 2023).

### Testicular histology

To assess testicular histology, testicular tissue were stained with hematoxylin and eosin (H&E), as described by Sarabia Fragoso et al. (2013). Immediately after euthanasia, a 1 cm^3^ piece of the left testes of each bird was cut and placed into 10% neutral buffered formalin for fixation. After 24 h, tissue samples were dehydrated (70, 80, 90, 96, and 100% ethanol), and cleared (pure xylenol) during a 16-h interval in the tissue processor (Model HS566 Carousel, China). After embedding tissues in paraffin, cross-sections (6 μm) were cut using a rotary microtome (LKB2218-020, Sweden). Slides were deparaffinized (xylenol), hydrated (ethyl alcohol 99.5, 90, and 80%, respectively), washed (water), stained in hematoxylin, floated in acid-alcohol and CaCO_3_ solution, stained in eosin, dehydrated (80, 90, and 99.5% ethanol, respectively), and clarified (xylenol).

**Fig 1:**
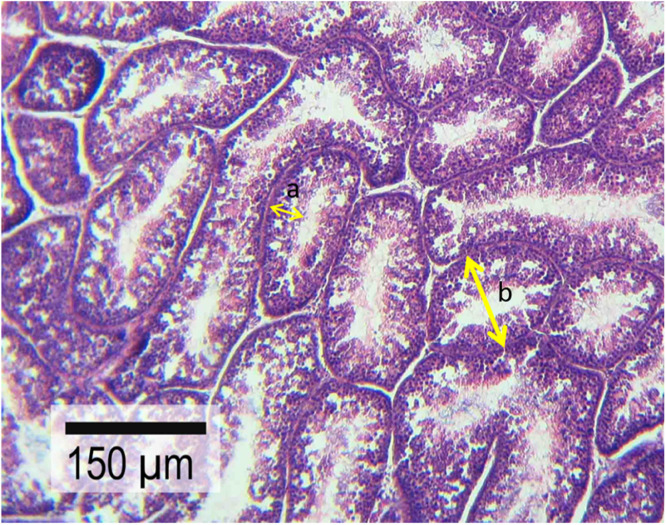
Light micrograph of the seminiferous tubules’ cross-section captured on a scale of 150 μm using a ×40 magnification of a light microscope (Olympus, BH2, Japan) equipped with a SONY on-board camera (Zeiss, Cyber-Shot, Japan). Arrows “a” and “b” indicate examples of sections that were considered to measure SET and STD, respectively, in testes tissue samples (samples were obtained at Week 12 of the experiment) for histological study.

**Fig 2:**
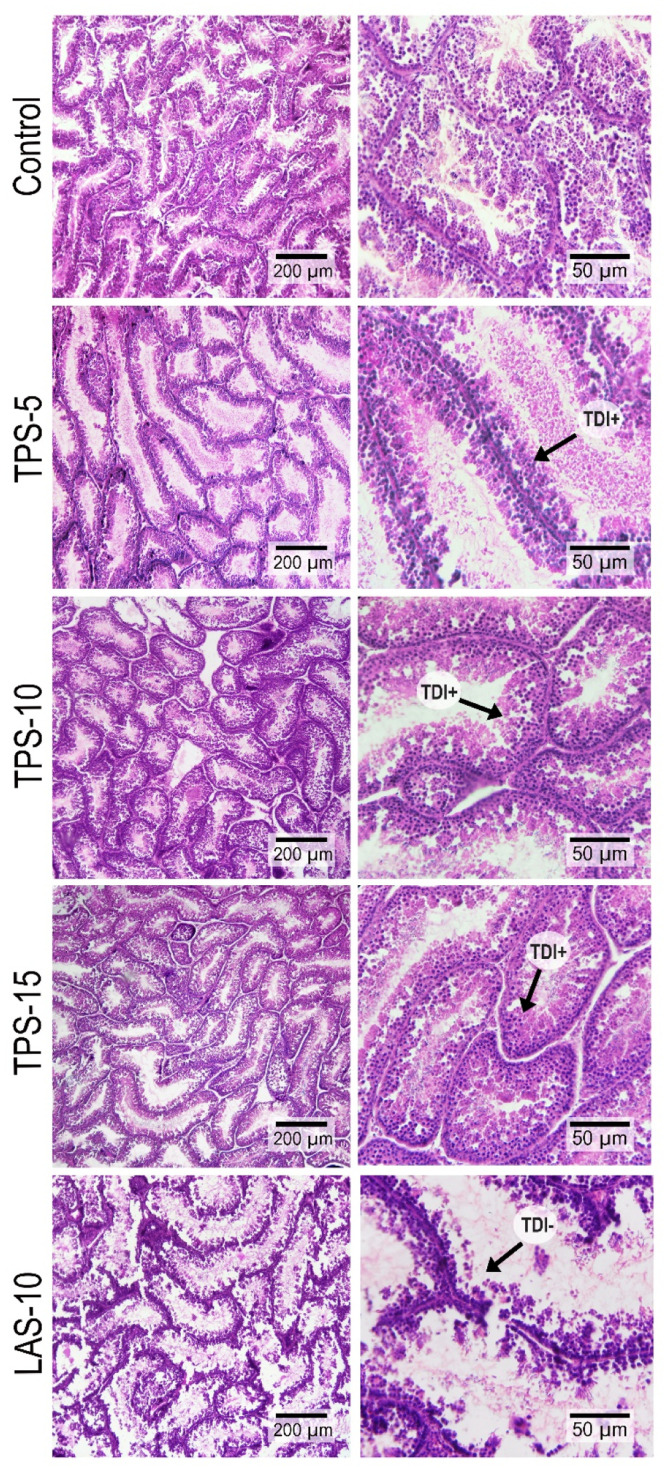
Light micrographs of the seminiferous tubules’ cross-sections (samples were obtained at Week 12 of the experiment) were captured on a scale of 200 and 50 μm using a ×40 objective lens on a light microscope (Olympus, BH2, Japan) equipped with a SONY on-board camera (Zeiss, Cyber-Shot, Japan).

Regarding testicular histology, STD and SET of at least 30 to 40 randomly selected round or semi-round cross-sections of regular seminiferous tubules were assessed (based on a micrometer) using a × 40 objective lens of a light microscope (Olympus, BH2, Japan) equipped with a SONY on-board camera (Zeiss, Cyber-Shot, Japan) with a scale set for 150 μm (Fig 1). Similarly, the TDI was evaluated by considering tubules with more than three or four germinal layers as positive TDI (Meistrich et al., 2003, Shetty et al., 2000) (Fig 2). Results for TDI analyses were reported as percentage of seminiferous tubules with negative TDI across the section.

### Gene expression

Quantitative mRNA expression of the *AKT1* and *Nrf2* genes in testes was evaluated according to the method of qRT-PCR at Rasta lab (West Azerbaijan Science and Technology Park, Urmia, Iran). Tissue samples were transferred to the laboratory at −196 °C and their mRNA extracted using the TRIZOL reagent, in line with manufacturer's instructions. The mRNA was quantified, and then the mixtures were diluted as follows: first, 1 λ of RNA from each sample was put on a Nanodrop (Maestrogen MN-913A, Taiwan) sensor, and light absorbance ratio at 260 to 280 nm determined. Then, diluted RNA samples with a concentration of 200 ng/μL were provided from the primer RNA samples. Finally, RNA samples were stored at −20 °C. Afterward, cDNA was synthesized using materials from Parstous (for cells and tissue; Iran) and mixed according to the manufacturer's instructions.

Primers were designed by Cinnagen Co. (Iran) diluted to a ratio of 1:9 using DEPC water, vortexed, and transferred into 0.2 ml microtubes. Primers (5´ to 3´) designation were as follows: *AKT1*F, CTACGGTGCGGAGATTGTGT (AT, 60 °C); *AKT1*R, CACAGCCCGAAGTCCGTTAT (AT, 60 °C); *Nrf2*F, ATGAGTCGCTTGCCCTGG (AT, 60 °C); *Nrf2*R CTTGTTTTCCGTATTAAG (AT, 60 °C); *GAPDH*F GGAGATTACTGCCCTGGCTCCTA (AT, 61 °C); *GAPDH*R GACTCATCGTACTCCTGCTTGCTG (AT, 61 °C). The reference clone (NCBI gene bank ID) and amplicon length (bp) for AKT1 were NM_395928 and 132; for Nrf2, they were NM_396014 and 194; and for GAPDH, they were NM_374193 and 73, respectively. On the last step of qRT-PCR, a PCR mixture containing 10 μL of 2 × Real-Time PCR Master Mix, 1 μL of forwarding primer (10 pmol), 1 μL of reverse primer (10 pmol), 10 ng template (cDNA), and DEPC water (amount required to obtain 20 μL of total solution for each sample) was provided and transferred into qRT-PCR (Pentabase-basetayper, Germany) after being centrifuged.

The experiment was conducted in three independent replicates. Time and temperature conditions of the qRT-PCR program were as follows: initial denaturing cycle at 95 °C for 10 min; a two-step cycle for denaturing (95 °C and 15 s for *AKT1, Nrf2*, and GADPH), and extension (60 °C for *AKT1* and *Nrf2*, and 61 °C for GADPH at 1 min); a three-step cycle for melt curve in the following order: 95 °C for 15 s, 75 °C for 1 min, and 95 °C for 15 s (respectively) for all genes, including GADPH. Relative quantities were determined by measuring fluorescence radiation increment as a result of dye binding, using AB Applied software. The threshold was adjusted to meet the fluorescence signals at the exponential phase after the reaction was conducted. Once the Cts were acquired, relative expression of desired genes was calculated according to the ΔΔCt method. The Kolmogorov-Smirnov test was used to normalize data distribution. The 2^-ΔΔCt^ of each observation was calculated, and mean values (for each treatment) were reported as fold change, with > 2 or <0.5 considered significantly different compared to the Control group.

### Statistical analysis

Data were analyzed using the GLM procedure of SAS 9.2 (SAS, 2009) software. Percentage data (FAs profile) was normalized by ArcSin√x transformation. This experiment was conducted based on a completely randomized design, with each rooster considered an individual experimental unit. The statistical model of Y_ij_ = μ + T_i_ + ε_ij_ was used (where Y_ij_ is the observation, μ the mean of observations, T_i_ the treatment effect, and ε_ij_ the experimental error of each observation). A Tukey test was used to determine differences between means and *p* <0.05 was considered significant. After verification of data normality based on the Kolmogorov-Smirnov test, gene expression data were analyzed using one-way ANOVA and a Tukey test in SAS 9.2. Graphs were prepared using GraphPad Prism 9.3.1 software (Prism, 2021) and values reported as mean ± SEM.

## Results

### Fatty acid profile of sperm

Sperm FA profile evaluation is in Table 3. Roosters in the L-10 group had the significantly highest methyl myristoleate (C14:1), palmitic acid (C16:0), oleic acid (C18:1n-9) and total saturated FAs. All TP-supplemented groups had the highest docosatetraenoic acid (C22:4 n-6) and total unsaturated FAs. There was a high docosahexaenoic acid (C22:6 n-3) and low n-6:n-3 ratio in T-10 and T-15 groups (*P*<0.05).

### Blood biochemical contents

**Fig 3:**
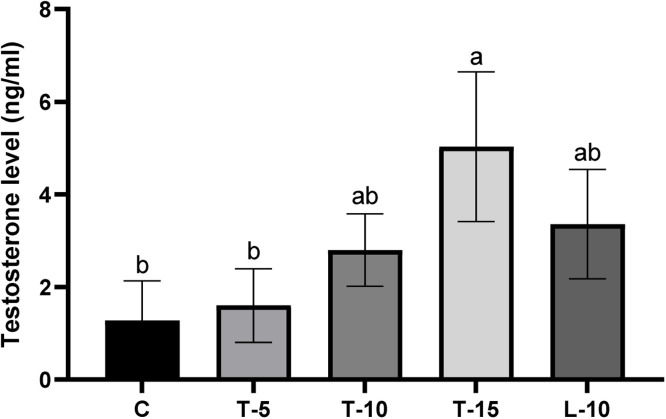
Effects of various dietary tomato pomace and L-Arg supplementation on serum testosterone concentrations (samples were obtained at Week 10 of the experiment) of aged male broiler breeders. Roosters (58 wk of age; *n*=6 per treatment) were fed a basal diet (C), or a basal diet supplemented with 5, 10 or 15% tomato (T-5, T-10 and T-15 respectively), or a diet supplemented with L-Arg 10% above the nutritional recommendation (L-10). Values represent mean ± SEM of blood samples collected from six birds per treatment. ^a,b^Means without a common superscript differed (*P*<0.05).

Serum concentrations of cholesterol and TG were highest in T-10 and lowest in T-15 (Table 4). The T-15 and L-10 groups had the highest TAC and UA and the lowest MDA, whereas the C group had the opposite. Furthermore, the T-5 group had the lowest AST compared to the C and L-10 groups (*P*<0.05). There were no significant differences among groups for serum concentrations of glucose, albumin or protein. Serum testosterone concentrations were highest in roosters fed T-15 (Fig. 3).

**Table 4:** Effects of dietary tomato pomace and L-Arg supplementation on blood biochemical components of aged male broiler breeders.

End point	C†	T-5	T-10	T-15	L-10	SEM	P-Value
Glucose (mg/dl)	177.00	192.66	197.00	211.33	201.00	10.320	0.278
Cholesterol (mg/dl)	103.33^bc^	116.33^ab^	127.66^a^	89.00^c^	112.66^ab^	3.621	0.001
TG* (mg/dl)	81.33^b^	110.66^ab^	146.66^a^	56.00^b^	78.33^b^	10.149	0.004
AST (IU/L)	122.66^a^	72.66^b^	97.33^ab^	93.00^ab^	105.33^a^	7.621	0.035
ALT (IU/L)	20	19.33	18	18	16.97	0.985	0.292
TAC (mmol/L)	1.346^b^	1.363^b^	1.356^b^	1.596^a^	1.763^a^	0.071	0.026
UA (mg/dl)	2.96^b^	3.29^b^	3.08^b^	6.51^a^	5.47^a^	0.614	0.039
Albumin (g/ml)	1.4	1.4	1.43	1.4	1.36	0.08	0.984
Protein (g/ml)	3.7	4.06	4.16	4.46	3.66	0.25	0.207
MDA (µg/ml)	3.20^a^	2.93^ab^	2.60^ab^	2.23^b^	1.63^b^	0.329	0.025

⁎ *Abbreviations:* TG, triglyceride; AST, aspartate amino transferase; ALT, alanine amino transferase, TAC, total antioxidant capacity; UA, uric acid; MDA, malondialdehyde; SEM, standard error of means.

a ^,b^ Means without a common superscript differed (*P*<0.05).

† Roosters (58 wk of age; *n*=6 per treatment) were fed a basal diet (C), or a basal diet supplemented with 5, 10 or 15% tomato pomace (T-5, T-10 and T-15 respectively), or a diet supplemented with L-Arg 10% above the nutritional recommendation (L-10). ^‡^Samples were obtained at Week 10 of the experiment.

### Testes biochemical contents

Regarding testicular biochemistry (antioxidant status), T-15 had the highest GPx compared to the C and L-10 groups, whereas the SOD was high in all experimental groups compared to group C (Table 5).

**Table 5:** Effects of dietary tomato pomace and L-Arg supplementation on testes biochemical components of aged male broiler breeders.

End point	C†	T-5	T-10	T-15	L-10	SEM	P-Value
TAC* (mmol/L)	0.25	0.37	0.29	0.32	0.24	0.032	0.091
GPx (U/mg protein)	4.49^b^	5.27^ab^	5.18^ab^	5.63^a^	4.43^b^	0.191	0.004
Protein (mg/dl)	148.66	163.00	151.66	156.00	157.66	4.626	0.293
SOD (U/mg protein)	5.51^a^	5.75^a^	5.86^a^	5.95^a^	4.21^b^	0.202	<0.001
MDA (U/mg protein)	0.21	0.16	0.17	0.16	0.18	0.016	0.177

⁎ *Abbreviations:* TAC, total antioxidant capacity; GPx, glutathione peroxidase; SOD, super oxide dismutase; MDA, malondialdehyde; SEM, standard error of means.

a ^,b^ Means without a common superscript differed (*P*<0.05).

† Roosters (58 wk of age; *n*=6 per treatment) were fed a basal diet (C), or a basal diet supplemented with 5, 10 or 15% tomato (T-5, T-10 and T-15 respectively), or a diet supplemented with L-Arg 10% above the nutritional recommendation (L-10)). ^‡^Samples were obtained at Week 12 of the experiment.

### Testes histology

The STP was increased in all TP-supplemented groups, whereas the SET was high in the T-10 and T-15 groups (*P*<0.05). The highest TDI values were in T-10 and T-15 groups whereas birds in the L-10 group had the lowest value among experimental groups (*P*<0.05).

### Gene expression

**Fig 4:**
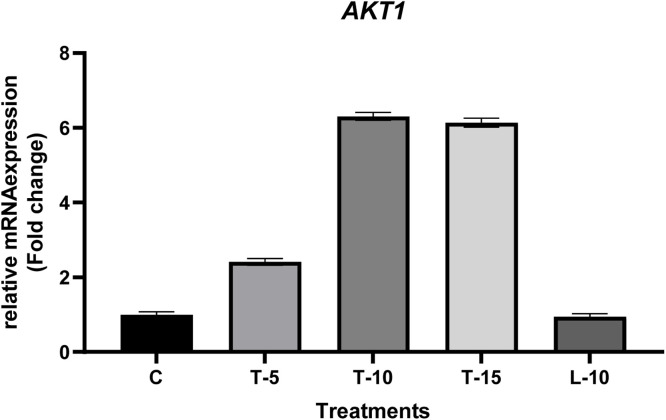
Relative mRNA expression of the *AKT1* gene. The data represent relative mRNA expression of *AKT1* in testicular tissue of aged male broiler breeders (samples were obtained at Week 12 of the experiment). Roosters (58 wk of age; *n*=6 per treatment) were fed a basal diet (C), or a basal diet supplemented with 5, 10 or 15% tomato (T-5, T-10 and T-15 respectively), or a diet supplemented with L-Arg 10% above the nutritional recommendation (L-10). Following the Kolmogorov-Smirnov normalize test and 2-ΔΔCt calculations, the mean values (for each treatment) were reported as fold change, and the treats with fold change > 2 or <0.5 considered significantly different compared to the control group.

**Fig 5:**
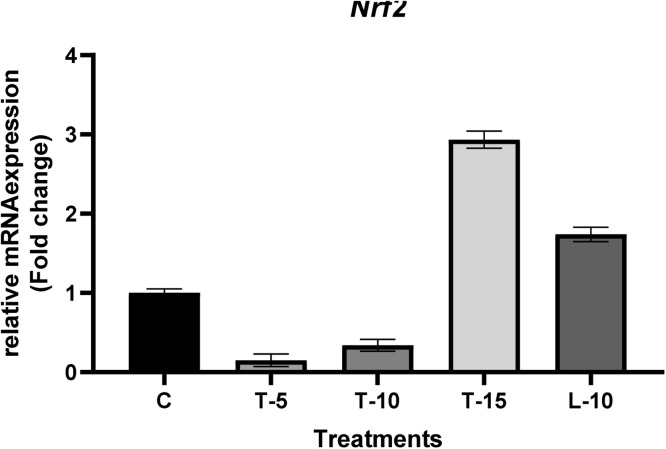
Relative mRNA expression of the *Nrf2* gene. Data represent relative mRNA expression of *Nrf2* in testicular tissue of aged male broiler breeders (samples were obtained at Week 12 of the experiment). Roosters (58 wk of age; *n*=6 per treatment) were fed a basal diet (C), or a basal diet supplemented with 5, 10 or 15% tomato (T-5, T-10 and T-15 respectively), or a diet supplemented with L-Arg 10% above the nutritional recommendation (L-10). Following the Kolmogorov-Smirnov normalize test and 2^-ΔΔCt^ calculations, mean values (for each treatment) were reported as fold change, and treatments with fold change > 2 or <0.5 were considered significantly different compared to the control group.

Effects of feeding TP and L-Arg supplemented diets on the relative mRNA expression of *AKT1* and *Nrf2* in testes tissue are shown in Fig 4, Fig 5, respectively. Relative mRNA expression of *AKT1* increased significantly in all TP-supplemented groups compared to both the C and L-10 groups (Fig 4). However, relative mRNA expression of *Nrf2* was reduced (*P*<0.05) in T-5 and T-10 groups (Fig. 5), but elevated in both the T-15 and L-10 groups (*P*<0.05).

## Discussion

The principal objective was to determine impacts of dietary TP and L-Arg supplementation on sperm FA profiles, biochemical components of serum and testes, testicular histology, and relative mRNA expression of the *AKT1* and *Nrf2* genes in testicular tissue of aged commercial male broiler breeders. This study followed our previous report (Mosayyeb Zadeh et al., 2023) that 15% dietary TP supplementation considerably improved sperm characteristics, reproductive performance, and ATP content and antioxidant status of semen in aged male broiler breeders; the current evaluations were conducted by using samples from birds in our previous study. The L-Arg supplemented diet (10% more than the nutritional recommendation) was included to determine whether the high L-Arg in TP content (see previous study) contributed to responses to TPS diets, or if those responses could be attributed mainly to lycopene.

Aging is the main fertility-reducing factor (Beckman and Ames, 1998) that disrupts the natural balance of antioxidant (especially lycopene) and ROS production and results in many diseases due to increased oxidative stress, including prostate cancer and cardiovascular diseases in humans (Petyaev, 2016). There are many suggested nutritional strategies to improve roosters’ fertility status, including dietary FA supplements, vitamin E and selenium, amino acids and their metabolites, probiotics, phytochemicals (including lycopene), plus feedstuffs or additives containing flavonoids and polyphenols, with a focus on antioxidants or antioxidant-related-activities (Fouad et al., 2020). Accordingly, effects of TP as a potential natural source of antioxidants and a by-product of commercial tomato paste factories was tested (in conjunction with L-Arg supplementation).

In the current study, dietary TPS or LAS altered whole-sperm FA composition. In general, unsaturated FA contents were increased in the T-15 group, whereas saturated FAs were increased in L-10. Although total saturated and unsaturated FA contents were not significantly different among sperm FAs of groups fed TPS or C, there was a remarkable reduction in n-6:n-3 ratio, C14:1, C16:0, and C18:1 n-9 and a considerable increment in C22:6 n-3 and C18:2 n-6 in the FA profile of the T-15-fed group compared to the C group. Unfortunately, FA content in rooster sperm was not evaluated in previous studies that investigated diets supplemented with TP or lycopene. Regardless, most of the present findings were in agreement with those of the previous investigations that used dietary apple pomace (Akhlaghi et al., 2014b), ginger rhizome (Akhlaghi et al., 2014a), or other dietary lipid sources (Bongalhardo et al., 2009), with a significantly high C22:6 n-3 and total unsaturated FA and significantly lower n-6:n-3 ratio and total saturated FA contents in sperm of roosters fed 20 to 25% apple pomace, 30 g ginger rhizome/kg, or fish oil supplemented diets, respectively.

Testes tissue sustains a consistent ratio of phospholipids in synthesizing sperm membranes, with phosphatidylcholine and phosphatidylethanolamine being dominant PLs (Blesbois et al., 1999; Blesbois et al., 1997; Kelso et al., 1996; Kelso et al., 1997a,b; Parks and Lynch, 1992). Despite a constant PL ratio, the FA composition of phosphatidylcholine and phosphatidylethanolamine was successfully changed by dietary oil supplementation from various sources, with n-3 FAs increased, and n-6:n-3 ratio decreased in sperm of birds fed fish or flaxseed oils (Bongalhardo et al., 2009). In contrast to plants, animals (especially birds) lack Δ-12 (n-6 FA producer) and Δ-15 (n-3 FA producer) desaturase, enzymes that convert dietary oleic acid into short-chain polyunsaturated FAs, such as linoleic acid (precursor of long-chain FAs, including EPA, DHA, etc.) (Ruiz-López et al., 2012, Venegas-Calerón et al., 2010). This accounts for earlier studies considering linoleic, alpha linoleic, and arachidonic acid as essential FAs in poultry nutrition (Balnave, 1970; Watkins, 1991).

However, recent studies reported the activities of Δ-five and Δ-six desaturase (expressed by FADS1 and FADS2 genes, respectively) (Cho et al., 1999a,b) and elongase 2 and elongase 5 (expressed by ELOVL2 and ELOVL5 genes, respectively) (Leonard et al., 2004; Wang et al., 2005) enzymes in the liver of male broiler chickens (Jing et al., 2013). Accordingly, Bongalhardo et al. (2009), referring to the formerly mentioned studies, declared that sperm membrane FAs are altered either directly or after a desaturation and elongation of dietary FAs, especially PUFAs (Blesbois et al., 1997; Walzem, 1996), in Sertoli cells (Retterstol et al., 2001). So, they assumed that dietary C18:2 n-6 may have desaturated and elongated to C20:4 n-6 in the roosters’ liver, and then, the obtained FA (C20:4 n-6) underwent a similar elongation process in testes to produce the C22:4 n-6 FA needed for sperm membrane synthesis (Cerolini et al., 1997).

Two compounds, C20:4 n-6 and C22:4 n-6, are the dominant FA in sperm membranes (Kelso et al., 1997b), and their association with flexibility and fluidity traits of sperm and the fertility status of roosters’ is well established (Surai et al., 2001). Therefore, in line with the study of Akhlaghi et al. (2014a), we concluded that the high C22:4 n-6 and total unsaturated FA contents in sperm membrane FAs of roosters fed T-15 may have contributed to numerical improvements in sperm motility of T-15 birds and significantly higher fertility compared to the C group (Mosayyeb Zadeh et al., 2023). Presumably the significantly high C14:1 and total saturated FA contents in the sperm membrane of bird fed L-10 reduced motility, viability, and fertility. Perhaps the Δ-five and Δ-six desaturase and elongase 2 and elongase 5 activities are also present in testes. Regardless, how lycopene or other bioactive compounds in TP affected these enzymes is not established and needs further investigation.

For by-products of commercial food-industry factories, e.g., tomato or apple pomace, the powerful antioxidant properties and relatively high CP and high CF prompt their use in animal nutrition. However, the exact mechanism of how these by-products in rooster diets can change the FA profile of rooster sperm is not clear. Improving antioxidant status in roosters (especially their semen) has been the main goal of various nutritional strategies (Fouad et al., 2020). For instance, supplementing (sub-fertile) male broiler breeders’ diet with phytochemicals, e.g., 30 mg/bird/day of turmeric (Kazemizadeh et al., 2019), 15 g/kg of ginger rhizome (Akhlaghi et al., 2014a), or feedstuffs with flavonoids and polyphenols, e.g., 5 g/kg of rosemary (Borghei-Rad et al., 2017), 20% dried apple pomace (Akhlaghi et al., 2014b), 30% TP (Saemi et al., 2012), or 250 ppm of cinnamon bark oil (Türk et al., 2015), improved roosters’ reproductive performance due to antioxidant properties.

Since TP is also a natural source of antioxidants, it was assumed that phenolic compounds in the TP used in the current experiment (895 mg/g gallic acid; [Mosayyeb Zadeh et al., 2023]), plus lycopene, could reach the testes and affect expression of genes involved in producing sperm itself, sperm FAs, antioxidant enzymes, etc. Although lycopene and total phenolic compound were relatively low in the current TP, improved antioxidant status of serum and testes (plus improved semen antioxidant status [Mosayyeb Zadeh et al., 2023]) in the present study were attributed to T-15 diets stimulating antioxidant responses, reducing lipid peroxidation, and protecting PUFA in sperm of broiler breeders. As reported in our previous study, numerically low MDA content in semen of roosters fed TPS diets (Mosayyeb Zadeh et al., 2023) may be good evidence for the above-mentioned effects of dietary TP and its lycopene. The antioxidative property of lycopene is 10 times more than α-tocopherol and double that of β-carotene (Giovannucci, 2002), due to: (i) its radical scavenging property, by delocalization of π electrons of its 11th conjugated double bond through the polyene chain, plus neutralization of ROS (Belter *et al.*, 2011)); and (ii) by its stimulatory impacts on expression of genes related to proteins with antioxidant or detoxifier activity, e.g., *Nrf2* (Gum and Cho, 2013; Taguchi et al., 2011). While confirming results from Saemi et al. (2012), who suggested that TP supplementation of up to 30% improved Iranian young native roosters’ reproductive performance, 15% dietary TP supplementation improved reproductive performance in aged roosters.

The antioxidative property of dietary TPS was implicated in protecting FAs against peroxidation and changing sperm FA profiles in the current study. In short, we inferred that an improved environment for Sertoli cells enhanced gene expression, promoted desaturase and elongase activities and resulted in fewer PUFAs peroxidized by ROSs. These assumptions were largely confirmed by biochemical assessments of serum and testes tissues (Table 4, Table 5) and that of semen in our previous study (Mosayyeb Zadeh et al., 2023). Therefore, high PUFAs in sperm of T-15 fed birds were attributed to protection by the antioxidant property of lycopene, whereas high SFAs in sperm of the L-10 group were due to high peroxidation of PUFAs by L-Arg-derived nitric oxide (**NO**). Most antioxidant indices, including TAC, UA, and MDA, were improved by T-15 or L-10 diets, which implied that T-15 and L-10 had common actions, presumably the antioxidant activity of their high L-Arg content. Metabolism of L-Arg in poultry increases NO (Kumar and Chaturvedi, 2008; Sundaresan et al., 2007) and guanidino acetic acid (Fouad et al., 2012), with antioxidant or radical scavenging activities. Therefore, high L-Arg (plus lycopene and other flavonoids and polyphenols in TP) likely improved antioxidant properties. Similarly, adding 10 mg/kg lycopene for 2 months in Wistar rats protected sperm and its DNA from oxidative-stress-induced varicocele (Babaei et al., 2021), implying lycopene had broad effects.

The T-15 diets remarkably reduced serum TG and cholesterol concentrations. These findings were in contrast to those of aged (65 wk) laying hens fed diets supplemented with up to 19% (190 g/kg) dried TP for 10 wk (Salajegheh et al., 2012). Apparent differences in responses were attributed to the birds’ strain and different nutritional requirements. However, apart from fish meal, the laying hens were also fed at least 1.6% (16 g/kg) dietary sunflower oil, whereas no fat was included in the diet of the current study. Regardless, the present results were in agreement with those of laying hens fed dietary supplements of turmeric (Mosayyeb Zadeh et al., 2022; Saraswati et al., 2013), sumac (Mosayyeb Zadeh et al., 2021), or Chinese herbal plant (Zhu et al., 2022), with substantial reductions in blood TG and cholesterol concentrations.

In birds fed bioactive compounds, it was expected that upregulation of lipolysis-promoting genes in their liver reduced blood TG concentrations (Zhu et al., 2022). Furthermore, the inhibitory impact of curcumin in turmeric on the Niemann‐Pick protein C1‐Like 1; NPC1L called protein (responsible for intestinal lipid absorption and biliary acids reabsorption) in enterocytes is responsible for low blood cholesterol (Feng et al., 2010). Therefore, consistent with previous studies, reduced serum TG and cholesterol were attributed to low intestinal absorption and high hepatic lipolysis due to TPs’ bioactive compounds (plus high dietary crude fiber).

Feeding TPS (especially T-5), significantly reduced serum AST, whereas serum AST in T-10 and T-15 groups tended to increase, though differences were not statistically significant compared to the C and L-10. For many years, AST and ALT have been used to identify hepatocyte injuries (McGill, 2016; Senior, 2012).

While confirming the theory of “oxidative stress theory of aging,” which discusses the oxidative stress-caused damage to the macromolecules (DNA, proteins, and lipids) by reactive oxygen and nitrogen spices (ROS and RON, respectively) accumulation due to aging (Beckman and Ames, 1998), these outcomes indicated that TP supplementation improved body (specifically testes) health status. Oxidative stress is identified by disruption of the natural balance between the antioxidant defense system and ROS production (Birben et al., 2012; Scandalios, 2006). Conversely, the natural decrease in lycopene due to aging is recognized as oxidative stress (Petyaev, 2016) and can result in prostate cancer or cardiovascular disease. In male Wistar rats, oral consumption of tomato seed oil (thrice weekly for 8 wk) protected tissues from gamma radiation-induced oxidative stress (by improving antioxidant status, reducing lipid peroxidation in testes and liver, and decreasing hepatic ALT and blood AST); these effects were attributed to antioxidant properties of its constituents, including lycopene, β-carotene, tocopherols, lutein, and phytosterols (Ezz et al., 2018). A significantly low AST in T-5 and the numerically low AST in T-10 and T-15 groups (compared to the C) of the current experiment indicated that body health status was threatened due to the aging-induced oxidative stress and that TPS could mitigate that threat by increasing lycopene (and antioxidants) and improving antioxidant and ROS production balance. In short, lycopene provided a healthier environment by its radical scavenging activity, so expression of genes (especially those related to sperm itself and sperm FA production, antioxidant enzymes, etc.) and protein activities were ameliorated.

Serum testosterone concentration of birds fed T-15 was significantly higher than those of the C and T-5 groups, but not statistically different compared to the T-10 and L-10 groups (Fig 3). Similarly, previous studies investigated effects of dietary natural antioxidants, including camphor (Raei et al., 2021), fish oil & rooibos (Golzar Adabi et al., 2023), sage extract (Ommati et al., 2013), and aqueous extract of zingiber (Saeid et al., 2011) on blood testosterone concentrations in male broiler breeders. In most previous studies, it was postulated that the increased blood testosterone was due to antioxidant impacts of bioactive compound on circulating luteinizing hormone (LH), which regulates testosterone secretion through binding to its receptors on Leydig cells (Vizcarra et al., 2000). As noted, the current test TP contained high L-Arg concentrations, the effects of which were observed in this experiment. Hence, compared to former attempts to study effects of dietary L-Arg supplementation on male broiler breeders’ reproductive performance (Abbaspour et al., 2019; Ahangar et al., 2017), and according to high testosterone concentrations in both the T-15 and L-10 groups, this finding was consistent with those in previous studies that maybe antioxidant and vasodilatory features of L-Arg-derived-NO increased blood flow to testes and therefore increased blood testosterone.

Conversely, lycopene can stimulate activity of NO-synthase enzyme (Palozza et al., 2012). Therefore, we inferred that probably the lycopene accompanied by the other phenolic compounds in TP may have stimulated testosterone secretion (by increasing circulating LH) in testes and the high testicular blood flow (due to high NO) may also have increased serum testosterone concentrations. In agreement with previous studies (Abbaspour et al., 2019; Ahangar et al., 2017), we believe that an increase in blood testosterone concentration (possibly due to high L-Arg or bioactive compounds like lycopene) may improve testes function by enhancing testicular-related parameters such as TDI, SET, STD, testes weight (not provided in the current manuscript because the data were not statistically significant), etc. This confirmed our previous assumption (Mosayyeb Zadeh et al., 2023), in which we, in agreement with Abbaspour et al. (2019) attributed the significantly high semen volume and sperm concentration in the T-15 group to increased serum testosterone.

In the testes of birds fed T-15 diets, both GPx and SOD were increased (significantly and numerically, respectively) compared to the C group. As well as the blood biochemical components discussed above, differences were attributed to antioxidant impacts of lycopene and its stimulatory effects on expression of enzymatic antioxidants’ genes, including *Nrf2*; expression of this gene regulates expression of antioxidant response element-mediated genes by binding to them (Shay et al., 2009). Therefore, expression of *Nrf2* is positively correlated with expression of genes encoding synthesis of enzymatic antioxidants, including GPx, SOD, etc. (Taguchi et al., 2011). This was confirmed by upregulation of *Nrf2* in testes tissue (Fig 5) and increased GPx and SOD content in semen in the present study. Both T-15 and L-10 up-regulated relative mRNA expression of the *Nrf2* gene; in line with previous investigations, it was believed that the stimulatory effect of lycopene was responsible for up-regulation of *Nrf2* expression in testes tissue (Gum and Cho, 2013; Taguchi *et al.*, 2011).

Relative mRNA expression of *Nrf2* was remarkably high in L-10 compared to the C, T-5, and T-10 groups; however, GPx and SOD in their seminal plasma were significantly (and TAC was numerically) lower than the other experimental groups. The exact mechanism is not clear and demands further investigations; however, we inferred that there may be a negative correlation between high NO production (through L-Arg metabolism) and low enzymatic antioxidants concentrations. Since NO is a highly reactive radical (Lorente et al., 1996) and its high concentrations may cause PUFAs peroxidation and stimulation of the cascade of reactions that increase ROS production (Semenova et al., 2005), enzymatic antioxidants may have mitigated free radicals, especially in semen (Mosayyeb Zadeh et al., 2023).

Neither live body weight nor testicular weight or size indices were affected by experimental treatments (data not reported), although STD, SET, and TDI were remarkably altered. To some extent, these findings contrasted to those reported by Abbaspour et al. (2019), who believed that perhaps the high blood flow to testes due to the L-Arg-derived NO (Bustos Obregón et al., 2006) increased left testis/total testicular weight index in the group treated with 0.68% L-Arg. They also reported that the height of epithelium seminiferous tubules or SET was not significantly changed by either L-Arg or flaxseed diets, inconsistent with current results. However, reduction in left testis/total testicular weight index and height of epithelium seminiferous tubules birds fed diets supplemented with higher levels of L-Arg (0.83%) and flaxseed (2%) (Abbaspour et al., 2019) was due to the apoptosis impact of high NO and ROS production in testes (El-Gohary et al., 1999). This was in agreement with STD, SET, and TDI of birds fed L-10 diets in the present experiment. Therefore, consistent with previously mentioned studies, the lowest STD, SET, and TDI values in the L-10 group (Fig 2), plus impacts on cell apoptosis and DNA damage were attributed to increased NO and ROS production.

That the STD, SET, and TDI indices increased significantly in testes tissue of birds in the T-10 and T-15 groups compared to those in the C were in complete agreement with studies that dietary selenium supplementation (Sabzian-Melei et al., 2022) or selenium + vitamin E (Khalid et al., 2016) increased STD and SET in aged male broiler breeders through their antioxidant features. The current discussion plus previous results implied that providing a healthier environment with an appropriate antioxidant level can enhance STD, SET, and TDI indices in rooster testes. Therefore, it was assumed that dietary TPS, especially T-10 and T-15, increased testicular STD, SET, and TDI due to the antioxidant property of its lycopene only, which is thought to affect gene expression by preventing ROS-caused DNA damage or via its stimulatory impact on expression.

In simple terms, in agreement with a previous study (Ye et al., 2021), we inferred that lycopene in the TP promoted healthier tissues, specifically testes (based on improved TAC, MDA, and enzymatic antioxidants quantities), and kept DNA and RNAs intact, which eventually enhanced relative mRNA expression of the genes such as *Nrf2, AKT1*, etc. This assumption was confirmed by up-regulation of the relative mRNA expression of the *AKT1* gene in the present experiment (Fig 4). Since *AKT1* contributes to spermatogenesis (Gao et al., 2019; Liu et al., 2019), we inferred that the antioxidant and stimulatory effects of TPs’ lycopene on up-regulation of *AKT1* increased TDI (Fig 2), and TDI in turn, increased STD and SET (Fig 1). As the TDI index assesses the percentage of seminiferous tubules containing three or more layers of type A spermatogonia, a higher TDI index directly leads to increased sperm production. Therefore, we concluded that the increased TDI, STD, and SET in the TP-supplemented groups (Table 6) were responsible for the higher sperm volume and concentration, as indicated in the previous study (Mosayyeb Zadeh et al., 2023), which alongside the high viable and high motile sperm indices contributed to improved fertility.

**Table 6:** Effects of dietary tomato pomace and L-Arg supplementation on testes morphology of aged male broiler breeders.

End point	C	T-5	T-10	T-15	L-10	SEM	P-Value
STD* (µm)	128.81^b^	151.93^a^	158.33^a^	159.03^a^	120.20^b^	4.023	<0.01
SET (µm)	20.12^b^	21.71^b^	28.21^a^	32.22^a^	18.76^b^	1.832	<0.01
TDI	38.45^b^	52.23^ab^	60.56^a^	66.27^a^	15.37^c^	4.176	<0.01

⁎ *Abbreviations:* STD, seminiferous tubular diameter; SET, seminiferous epithelium thickness; TDI, tubular differentiation index; SEM, standard error of means.

a ^-c^Means without a common superscript differed (*P*<0.05). ^†^Roosters (58 wk of age; *n*=6 per treatment) were fed a basal diet (C), or a basal diet supplemented with 5, 10 or 15% tomato (T-5, T-10 and T-15 respectively), or a diet supplemented with L-Arg 10% above the nutritional recommendation (L-10). ^‡^Samples were obtained at Week 12 of the experiment.

## Conclusion

In the T-15 group, the stimulatory impact of TPs’ lycopene on expression of antioxidant genes improved antioxidant status in serum and testes; that, plus its unique radical scavenging property (and perhaps L-Arg derived NO and guanidino acetic acid) provided a healthier environment for up-regulation of genes supporting spermatogenesis, e.g., *AKT1*. These events are thought to have altered sperm FA profile (increased PUFAs and decreased n-6:n-3) and increased STD, SET, and TDI. Furthermore, high serum testosterone concentrations in T-15-fed birds were attributed to antioxidant and vasodilatory impacts of lycopene and L-Arg derived NO, respectively. We postulated that the improved fertility status of T-15 fed roosters (in the previous study) were due to the TPs’ lycopene and high L-Arg contents that enhanced antioxidant status, up-regulated spermatogenesis genes, and improved sperm FA profile and semen characteristics. Based on the current outcomes, up to 15% TP supplementation is recommended to improve reproductive performance by enhancing testes morphology and antioxidant status in aged male broiler breeders.

## Funding

This research did not receive any specific grant from funding agencies in the public, commercial, or not-for-profit sectors.

## Declaration of competing interest

The authors declare that they have no known competing financial interests or personal relationships that could have appeared to influence the work reported in this paper.
